# Experimental Uremia

**Published:** 1882-02

**Authors:** 


### EXPERIMENTAL UREMIA.

A recent work published by MM. Feltz and Ritter (Paris, 1881) summarizes the results of researches which they have carried on for the last fifteen years, and it is analyzed in an interesting review by M. Lereboullet, in the *Gazette Hebdomadaire*. . . . They have laboriously endeavored to ascertain what is the influence of the injection into the blood of principles which the organs of excretion eliminate. They have proved that the words cholemia and cholesteremia, do not at all mean what those who introduced them into the scientific language thought them to mean. The injection into the blood of the coloring matters of bile and of cholesterine, even of the biliary acids, never excite symptoms of grave icterus.

In investigating the result of the injection of the substances known as extractives, the injection of various ammoniacal salts, or of those of urea, they have attacked the theory of ammonemia supported by Virchow, and that of Schottin, and have demonstrated that it was only in an impure state that urea excited the convulsions which have caused certain physiologists to put forward the theory of uremia. But the most remarkable result of their patient researches is that of the intravenous injection of fresh or stale urine. The injection into the vein of a dog of fresh human urine, filtered and warmed to 35°C., (95° F.) very rapidly excites symptoms identical with those which characterize the uremia determined by ligature of the ureter or of the renal vessels, that is to say, by sudden suppression of the renal function.

These uremic symptoms are not due either to the augmentation of intravascular tension or to acidity of the urine, since injections made with pure or with acidulated water are harmless. But—and this is the essential fact of these new researches—they are likewise not due to the introduction into the veins of the organic matters of the urine. These, when injected either alone or together, do not possess the symptoms of uremia, which absolutely contradict the theories of Wilson and Schottin. On the other hand, by introducing into the blood the mineral salts contained in urine three days old, the authors have reproduced exactly the same phenomena as by acting with fresh normal urine or urine strongly concentrated by repeated coagulation. Pursuing their very interesting researches MM. Feltz and Ritter observed that the poisonous salts of the urine were the salts of potash ; that the same uremic symptoms were excited by injecting into the blood normal urine as by introducing the salts of potash dissolved in distilled water in proportions equal to those which this urine contained. If clinical observation should confirm the experiments made on animals, and it be therefore demonstrated that uremia is nothing else than poisoning by salts of potash accumulated in the blood or fixed in excess in the tissues, great progress will have been made ; nor would it be rash to suppose that such a discovery would put the physician on the path of new therapeutical methods applicable to the treatment of uremic symptoms.—*British Med. Journal.*

